# Diagnosis and Management of Hematological Adverse Events Induced by Immune Checkpoint Inhibitors: A Systematic Review

**DOI:** 10.3389/fimmu.2020.01354

**Published:** 2020-10-21

**Authors:** Nabil E. Omar, Kareem A. El-Fass, Abdelrahman I. Abushouk, Noha Elbaghdady, Abd Elmonem M. Barakat, Ahmed E. Noreldin, Dina Johar, Mohamed Yassin, Anas Hamad, Shereen Elazzazy, Said Dermime

**Affiliations:** ^1^Pharmacy Department, National Center for Cancer Care and Research, Hamad Medical Corporation, Doha, Qatar; ^2^Department of Pharmacy Practice, College of Clinical Pharmacy, King Faisal University, Hofuf, Saudi Arabia; ^3^Division of Cardiology, Department of Medicine, Harvard Medical School, Boston, MA, United States; ^4^Clinical Pharmacy Department, School of Pharmacy, New Giza University, Giza, Egypt; ^5^Faculty of Veterinary Medicine, Damanhour University, Damanhour, Egypt; ^6^Department of Histology and Cytology, Faculty of Veterinary Medicine, Damanhour University, Damanhour, Egypt; ^7^Basic Sciences Department, Faculty of Medicine, Algalala University, Suez, Egypt; ^8^Medical Oncology-Hematology Section, National Center for Cancer Care and Research, Hamad Medical Corporation, Doha, Qatar; ^9^National Centre for Cancer Care and Research, Hamad Medical Corporation, Doha, Qatar

**Keywords:** immune checkpoint inhibitors, immune-related adverse events, ipilimumab, pembrolizumab, nivolumab, atezolizumab, durvalumab, avelumab

## Abstract

There has been less volume of literature focusing on the Immune-related Hematological Adverse Drug Events (Hem-irAEs) of Immune Checkpoint Inhibitors (ICPis) in cancer patients. Furthermore, there has been no consensus about the management of hematological toxicity from immunotherapy in the recently published practice guidelines by the European Society for Medical Oncology (ESMO). We conducted a systematic review of case reports/series to describe the diagnosis and management of potentially rare and unrecognized Hem-irAEs. We searched Medline, OVID, Web of Science for eligible articles. Data were extracted on patient characteristics, Hem-irAEs, and management strategies. We performed quality assessment using the Pierson-5 evaluation scheme and causality assessment using the Naranjo scale. Our search retrieved 49 articles that described 118 cases. The majority of patients had melanoma (57.6%) and lung cancer (26.3%). The most common Hem-irAEs reported with ICPis (such as nivolumab, ipilimumab, and pembrolizumab) were thrombocytopenia, hemolytic and aplastic anemias. Less reported adverse events included agranulocytosis and neutropenia. Steroids were commonly used to treat these adverse events with frequent success. Other used strategies included intravenous immunoglobulins (IVIG), rituximab, and transfusion of blood components. The findings of this review provide more insights into the diagnosis and management of the rarely reported Hem-irAEs of ICPis.

**Graphical Abstract:**
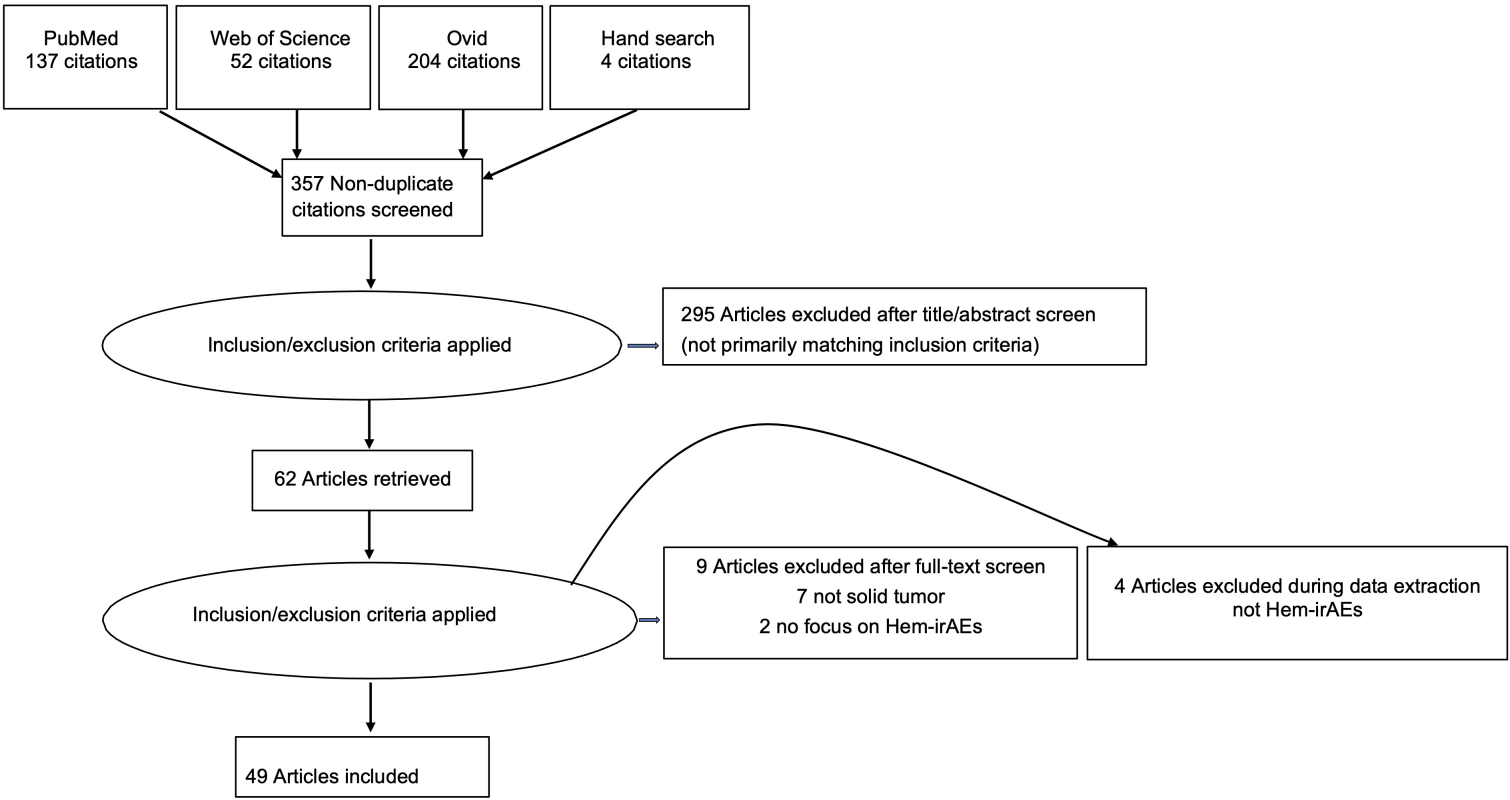
The flow of the article selection process is presented as PRISMA.

## Introduction

In the past decade, the enthusiasm for connecting the immune system and malignancy has expanded. Exploiting the host's immune system to treat cancers depends on immune surveillance: the ability of the immune system to identify foreign neo-antigens and target them for elimination (1). Immune checkpoint receptors, i.e., cytotoxic T-lymphocyte-associated protein 4 CTLA4 antibody ipilimumab, and programmed cell death protein-1 (PD-1) are critical for the physiological responses of the immune system. Checkpoint signaling triggers immune tolerance of T-cell activation to avoid autoimmunity and the adverse effects of excessive inflammatory responses. Tumor cells utilize these mechanisms to avoid destruction by the immune system (2).

In August, 18, 2010, the FDA approved the CTLA-4 ipilimumab antibody as the first ICPi for the treatment of metastatic melanoma (3). The filing was based on results from the primary analysis of the pivotal MDX010-020 trial, which were published online in the New England Journal of Medicine and presented in June 2010 during a plenary session at the 46th Annual Meeting of the American Society of Clinical Oncology (3). Despite its approval, ICPis have not been widely used except in the last 2 years. Recently, PD-1 inhibitors were approved for the treatment of non-small cell lung cancer (NSCLC) (4). Following their approval, these immunotherapeutics became integral parts of the treatment protocols against melanoma and NSCLC. Furthermore, they have shown promising responses [objective response rates (ORRs)] against different cancers, including mismatch repair deficient colorectal cancer (60%) and Hodgkin's disease (65–85%) (5).

Although the side effects of immunotherapy are less than chemotherapeutic agents (4), immunotherapy still may cause dermatological (reticular, maculopapular erythematous rash, and mucositis), gastrointestinal (diarrhea and colitis), hepatic (elevation of liver enzymes in serum), and endocrine adverse effects (involving pituitary, adrenal, or thyroid glands). This is because the immune response triggered by these drugs is not completely tumor-specific (6). The management of their adverse events usually includes various forms and regimens of corticosteroids (7).

With the expanding use of ICPis in clinical practice, more rare side effects are being discovered. Some Hem-irAEs were described, including immune thrombocytopenia, autoimmune hemolytic anemia, agranulocytosis, or pure red cell aplasia (8). The evidence focusing on the Hem-irAEs of ICPis is scarce. Moreover, there is no consensus on the management of hematologic toxicity from immunotherapy in the recently published practice guideline by ESMO (9). We aimed to evaluate the published literature on this topic and summarize the successful management approaches of the rare side effects.

## Methods

### Data Sources and Searches

We commenced this study in May 2018 and included all available updates published since 2008 till the present time.

We conducted literature search using different databases: Medline, OVID, and Web of Science. Furthermore, we searched the gray literature; conference proceedings; using Web of Conferences, Open Grey up to January 2019. We searched the bibliographies of relevant studies for any eligible case reports/series up to January 2019. The flow of the article selection process is presented in the graphical abstract as Preferred Reporting Items for Systematic Reviews and Meta-Analyses (PRISMA) figure. We used no time limit to date.

We used well-defined keywords. The search terms are listed in Appendix 1. The following keywords: (immune checkpoint inhibitors), (ICPis), (immunotherapy) (ipilimumab), (programmed cell death), (Programmed Cell Death 1 Receptor), (Programmed death ligand), (pembrolizumab), (nivolumab), (atezolizumab), (durvalumab), (avelumab) (adverse drug reaction), (adverse effects) (hematological adverse effect), Immune related adverse event (pancytopenia), (immune thrombocytopenic purpura), (thrombocytopenia), (leucopenia), (anemia) and (neutropenia) were entered, and the search was limited to articles in English. A summary of the 49 enrolled studies, clustered based on the medication used and Hem-irAEs experienced is shown in Table 1.

**Table 1:** Summary of available literature about immune check point inhibitors-associated hematological adverse effects.

**References**	**Therapeutic agent**	**Diagnosis**	**Number of cases**	**Hematological adverse effect/s**	**Occurred after how many cycles/days post ICPis**	**Intervention or management of hematological adverse effect/s**	**Outcome of hematological adverse effect/s management**
(10)	Pembrolizumab	Metastatic melanoma	Case A	Immune thrombocytopenia	A: 1st cycle	A: three boluses of methylprednisolone and two infusions of immunoglobulins (2 g/kg). Followed by oral corticosteroid therapy then tapered down B: a course of corticosteroid was initiated (1 mg/kg/d)	Resolved Resolved
			Case B		B: NA		
(11)	Pembrolizumab	Metastatic melanoma	1	Immune thrombocytopenia	After the 2nd dose of pembrolizumab	Steroids	Ineffective
(12)	Pembrolizumab	Metastatic melanoma	1	Pancytopenia	The 18th cycle	High dose prednisolone and a 5 day course of IVIG therapy	Resolved after IVIG course
(13)	Pembrolizumab	Metastatic melanoma	1	Warm antibody autoimmune hemolytic anemia and pure red cell aplasia	The 3rd cycle	High dose glucocorticoids	Pure red cell aplasia flared when prednisone tapered to 20 mg Subsequent treatment with one dose of IVIG enabled tapering of the glucocorticoids
(14)	Pembrolizumab	Stage 4 lung adenocarcinoma	1	Sever neutropenia	The 2nd cycle	G-CSF, IV solumedrol, IVIG, cyclosporine A	Recovered
(15)	Pembrolizumab	Metastatic bladder cancer	1	Hemophagocytic lymphohistiocytosis	NA	Etoposide and dexamethasone	NA
(16)	Pembrolizumab	Metastatic NSCLC	1	Evan's syndrome	After the 18th cycle	Pembrolizumab discontinuation and prednisone, azathioprine, cyclophosphamide, and IVIG therapy combined with erythropoietin injections and transfusion, then weekly rituximab and re-initiation of high dose prednisone	Resolved
(17)	Pembrolizumab	Stage 3a lung adenocarcinoma	1	Exacerbation of autoimmune hemolytic anemia	17 days after the 1st cycle	IV steroids and blood transfusion	Recovered but patient died 33 days later
(18)	Pembrolizumab	Metastatic melanoma	1	Autoimmune hemolytic anemia	The 4th cycle	IV steroids	Recovered
(19)	Pembrolizumab	Metastatic melanoma	1	Autoimmune hemolytic anemia	The 3rd cycle	Steroids, rituximab and pembrolizumab discontinuation	Resolved
(20)	Nivolumab	Metastatic melanoma	1	Severe anemia and thrombocytopenia (Bicytopenia)	The 6th cycle	RBCs, platelet transfusion and high dose IV methylprednisolone	Ineffective
(21)	Nivolumab	Metastatic NSCLC	1	Severe pancytopenia	After the 3rd cycle	IV steroids, G-CSF and IVIG	Ineffective
(22)	Nivolumab	Metastatic NSCLC	1	Exacerbation of underlying immune thrombocytopenia	After the 9th cycle	IV romiplostim, withholding of nivolumab	Recovered and nivolumab resumed
(23)	Nivolumab	Metastatic NSCLC	1	Immune Thrombocytopenia	After the 6th cycle	Discontinuation of nivolumab, platelet transfusions were given for 4 weeks then IV steroids	Resolved
(24)	Nivolumab	Metastatic NSCLC	1	Immune-mediated thrombocytopenia and hypothyroidism	After the 2nd cycle	IV steroids, levothyroxine and discontinuation of nivolumab	Recovered
(25)	Nivolumab	Metastatic melanoma	1	Severe thrombocytopenia, ITP	Before the 3rd dose	Prednisolone, IVIG, romiplostim and platelet transfusion	Resolved
(4)	Nivolumab	Metastatic NSCLC	1	Severe agranulocytosis	The 2nd cycle	3 doses of IVIG without improvement, then oral 1.5 mg/kg/day prednisone for 3 days without improvement, count improved after high dose IV methylprednisolone	Resolved only after high dose methylprednisolone (3 mg/kg IV)
(26)	Nivolumab	Metastatic NSCLC	Case A Case B	Severe complicated neutropenia	Case A: the 5th cycle Case B: after the 9th cycle	Case A: G-CSF, IV steroids Case B: G-CSF, IV steroids	Case A: ineffective and patient passed away 13 days later Case B: ineffective
(8)	Nivolumab	Stage IV adenocarcinoma of the lung	Case A Case B Case C	Bone marrow failure as an immune-related aplastic anemia	NA	A: IVIG, antibiotics 4 RBCs units, and 3 platelets units B: prednisone 1 mg/kg, norethandrolone, G-CSF, 4 RBCs and 9 platelets units C: prednisolone 1 mg/kg IVIG, G-CSF, antibiotics, 20 RBCs and 15 platelets units	A: no response to IVIG, death at 1 month of febrile neutropenia B: partial and transient response to steroids, persistent pancytopenia ongoing at 4 months C: no response to steroids and IVIG, death at 3 months from acute coronary syndrome
(27)	Nivolumab	Metastatic melanoma	1	Symptomatic warm autoimmune hemolytic anemia	The 4th cycle	Discontinuation of nivolumab and prednisone	Resolved
(28)	Nivolumab	Metastatic cutaneous squamous cell carcinoma and CLL	1	Hemolytic anemia	The 8th cycle	Discontinuation of nivolumab and prednisone	Anemia recovered after 2 weeks
(29)	Nivolumab	Stage 4 lung adenocarcinoma	1	Autoimmune hemolytic anemia	The 2nd cycle	Prednisolone	Ineffective
(30)	Nivolumab	Glioblastoma multiforme	1	Aplastic anemia	After the 2nd cycle	G-CSF, eltrombopag and blood transfusion	Ineffective, death 73 days after the 2nd dose of nivolumab
(31)	Nivolumab	Metastatic melanoma	1	Pure red cell aplasia	The 31st cycles	IV steroids and blood transfusion, nivolumab was discontinued	Recovered
(32)	Nivolumab	Metastatic melanoma	1	Severe allograft rejection and autoimmune hemolytic anemia	NA	IV steroids	Recovered
(33)	Nivolumab	Stage 4 NSCLC	1	Immunotherapy-associated hemophagocytic syndrome	After the 2nd dose	IV steroids	Resolved with tumor regression
(34)	Nivolumab	Metastatic lung squamous cell carcinoma	1	Acquired hemophilia A	After 17 months from the 1st cycle	Oral steroids then IV cyclophosphamide and factor VII	Resolved
(35)	Ipilimumab	Metastatic melanoma	1	Autoimmune pancytopenia	8 days after the 4th cycle	High dose corticosteroids Erythropoietin 30,000 IU/wk, N-plate 1 mg/kg/wk, filgrastim 10 mg/kg/d and IVIG	Pancytopenia was resistant to high dose oral corticosteroids and to hematopoietic growth factors, but resolved after IVIG injection
(36)	Ipilimumab	Metastatic melanoma	1	Pancytopenia	After 36 weeks	Growth factors, transfusions, antibiotics, immunoglobulins, and immunosuppressive therapy (cyclosporine)	Ineffective
(37)	Ipilimumab	Metastatic melanoma	1	Pancytopenia with cerebral hemorrhage and respiratory insufficiency	Unknown	Steroids	Ineffective
(38)	Ipilimumab	Metastatic melanoma	1	thrombocytopenia	Day 12 after the 2nd cycle	1 mg/kg prednisolone and 1 g/kg IVIG	Resolved
(39)	Ipilimumab	Metastatic melanoma	1	Immune-mediated thrombocytopenia.	After the 1st cycle	IV steroids, platelet transfusion, oral steroids and ipilimumab discontinuation	Effective
(40)	Ipilimumab	Metastatic melanoma	1	Acute grade 4 neutropenia	14 days after the 4th cycle	CSF, steroids and IVIG	Neutropenia did not respond to CSF and steroids, it rapidly improved after administration of IVIG
(41)	Ipilimumab	Metastatic melanoma	1	Febrile neutropenia with agranulocytosis	14 days after administration of the 3rd cycle	Filgrastim, meropenem, fluconazole IV, and 2 mg/kg of methylprednisolone (120 mg) IV daily, and was discharged on 128 mg oral methylprednisolone daily	Ineffective
(42)	Ipilimumab	Metastatic melanoma	Case A Case B Case C	A: hemolytic autoimmune anemia B: severe leukopenia and febrile neutropenia C: severe anemia and leukopenia	A: after the 3rd cycle B: after the 3rd cycle C: after treatment discharge (48 weeks from initial dose), during follow up	A: high dose methylprednisolone and blood transfusion B: antibiotics, GM-CSF and high doses of IV Methylprednisolone followed by tapering C: oral corticosteroids prednisone 1 mg/kg/day and GM-CSF for 1 week	A: resolved B: resolved C: resolved
(43)	Ipilimumab	Stage IIIB melanoma	1	Neutropenia	After the 4th cycle	- Oral steroids, - IV cyclosporine, - IVIG, - G-CSF, - IVATG	Resolved after 7.5 weeks from the 4th dose
(44)	Ipilimumab	Metastatic melanoma	1	Large granular lymphocytosis with severe neutropenia	After the 3rd cycle	Discontinuation of ipilimumab, IV antibiotics, G-CSF, IVIG, IV steroids, IVATG, IV cyclosporine	Resolved after IVATG plus cyclosporine and steroids
(45)	Ipilimumab	Metastatic melanoma	1	Acquired hemophilia A	After the 3rd cycle	IV steroid, factor VII and tranexamic acid	Effective, bleeding stopped
(46)	Ipilimumab	Metastatic melanoma	1	Immune-mediated red cell aplasia	After the 9th cycle	Oral prednisone at 1 mg/kg /day with little change in his transfusion requirement after 4 weeks, he received IVIG	Poor response to corticosteroids and rapid clinical benefit from IVIG
(47)	Ipilimumab	Metastatic melanoma	1	Hemophagocytic syndrome	After the 2nd cycle	IV steroids and IV etoposide	Ineffective
(48)	Durvalumab	NSCLC	1	A fatal allo- and immune-mediated thrombocytopenia	Two months after cessation of treatment with the PD-L1 inhibitor	Platelet transfusion daily for 12 days and polyvalent immunoglobulins (25 g/day for 4 days) and steroid treatment (1 mg/kg)	No improvement and death occurred 36 days after the 1st transfusion due to intra-alveolar hemorrhage
(49)	Avelumab	Metastatic Merkel cellcarcinoma	1	Lethal thrombocytopenia	After the 4th cycle	IV steroids, IVIG	Ineffective, patient died 1 month of ITP
(50)	Ipilimumab and nivolumab	Case A: melanoma stage IIb Case B: metastatic melanoma	Case A Case B	Severe thrombocytopenia	A: The 1st cycle B: 43 days after nivolumab monotherapy and 8 days after ipilimumab monotherapy	A: 1st dose of steroids and IVIG, then rituximab B: prednisone, IVIG, and rituximab, cessation of ipilimumab	A: no response to steroids or IVIG, recovered after 4 doses of rituximab B: Resolved
(51)	Ipilimumab plus nivolumab	Metastatic melanoma	1	Aplastic anemia	After four courses of the combined treatment, followed by five courses of nivolumab in 3 days	Daily treatment with prednisone (1 mg/kg), and G-CSF	At the 11th day of hospitalization patient suffered brain hemorrhage with rapid fatal outcome
(52)	Ipilimumab and nivolumab	Metastatic melanoma	1	Autoimmune hemolytic anemia	The 2nd cycle	Multiple blood transfusions and started on pulse dose steroids using 1,000 mg of IV methylprednisolone daily for 3 days then course of oral prednisone, had AHA after re-challenging with immunotherapy which responded faster to rituximab	First occurrence responded gradually to corticosteroid Due to slow response to steroids after the 2nd occurrence of AHA; rituximab added, and the patient responded well to it
(53)	Case A: ipilimumab Case B: pembrolizumab Case C: pembrolizumab Case D: ipilimumab and nivolumab	A: prostate cancer B: metastatic melanoma C: SCLC D: metastatic melanoma	A B C D	A: neutropenia B: hemolytic anemia C: hemolytic anemia D: hemolytic anemia	A: after the 2nd cycle of ipilimumab B: After 3 weeks of immunotherapy C: After 2 weeks of pembrolizumab D: on day 33	A: Methylprednisolone at 1 mg/kg every 12 h IV for 3 consecutive days and subsequent oral prednisone at 1 mg/kg daily B: IV methylprednisolone 1 mg/kg once daily for 3 days and then transitioned to oral prednisone 1 mg/kg daily for 2 additional weeks C: prednisone at 1 mg/kg/d D: prednisone 1 mg/kg/d initially which was increased to 2 mg/kg/d after day 38 when platelet count dropped to 5,000/μL IVIG 1 g/kg/d for 2 days for presumed immune thrombocytopenia	A: Resolved B: Resolved C: Resolved D: Resolved
(54)	Pembrolizumab (*n* = 17), nivolumab (*n* = 7), and durvalumab (*n* = 2)	Melanoma (*n* = 20), renal cell carcinoma (*n* = 3), other tumor types (*n* =3)	26	Increase in AEC	After a median of 3.0 months after the 1st cycle	NA	NA
(55)	Ipilimumab and nivolumab	Metastatic melanoma	1	Aplastic anemia	Two weeks following the 2nd cycle	IV methylprednisone 70 mg/ day for 8 days, followed by a prednisone taper.	Recovery
(56)	Nivolumab (*n* = 20), pembrolizumab (*n* = 14), and atezolizumab (*n* = 1)	Melanoma (*n* = 15), NSCLC (*n* = 12), and other types of cancers (*n* = 8)	35	Neutropenia 9 (26%), anemia 9 (26%), thrombocytopenia 9 (26%), pancytopenia or aplastic anemia 5 (14%), bicytopenia 2 (6%), and pure red cell aplasia 1(3%)	Median time to onset was 10.1 weeks	22 (63%) of 35 patients were given steroids orally, 5 (14%) were given steroids IV and orally, 11 (31%) had IVIG, and 7 (20%) had rituximab	21 (60%) of patients recovered

*ICPi, Immune Check Point inhibitors; IVIG, Intravenous Immunoglobulin; IVATG, Intravenous Anti-thymocyte Globulin; CSF, Colony Stimulating Factor; G-CSF, Granulocyte Colony Stimulating Factor; GM-CSF, Granulocyte-Macrophage Colony Stimulating Factor; RBC, Red Blood Cells; NA, Not Available; ITP, Idiopathic Thrombocytopenic Purpura; SCLC, Small Cell Lung Carcinoma; AEC, Absolute Eosinophil Count; NSCLC, Non-Small Cell Lung Carcinoma; AHA, Autoimmune Hemolytic Anemia*.

Initial screening of the eligible articles was done independently by two authors NO and NE. The articles were screened first based on their titles and abstracts, and then the full text was reviewed to decide the eligibility. Any conflict was solved by a third author KE. Only full-text articles published in peer-reviewed journals were retrieved for review according to the following criteria. AA, MY, AH, SE contributed to data analysis.

#### Inclusion Criteria

1. Case reports/series of solid tumors;
2. Reporting Hem-irAEs;
3. Using ICPis, monotherapy or combinations either as part of a clinical trial or during clinical practice;
4. English language;
5. Adults or pediatrics.

#### Exclusion Criteria

6. Other irAEs than Hem-irAEs;
7. Non-solid tumors;
8. Article reporting side effects which are not immune related;
9. Use other medications than ICPis causing Hem-irAEs;
10. Use of non-FDA approved ICPis up to the date of data extraction.

#### Data Extraction and Quality Assessment

Data was extracted by NO and NE, then was revised by KE. The extracted data included type of cancer, ICPis, number of cases, Hem-irAEs, onset of the adverse events, management of Hem-irAEs, and management outcomes. We used the Pierson-5 evaluation scheme (57) to assess quality of case reports based on 5 domains: documentation, uniqueness, educational value, objectivity, and interpretation as shown in Table 2. Each domain is scored, for example (0, 1, or 2 points, the upper score is 10 points). When a case report scores 9–10 points, the report contributes to the literature; a 6–8 points indicates validity and clinical value of the report are doubtful; a 5 points or less indicates insufficient quality. The assessment was carried out by KE and a random sample was crosschecked by NO and NE.

**Table 2:** Number of case reports with different scores for the five domains of Pierson-5 scale.

Domain/score	0	1	2
Documentation	3	9	42
Uniqueness	12	23	19
Educational value	3	29	22
Objectivity	1	19	34
Interpretation	1	8	45

#### Causality Assessment

Each case report was assessed according to Naranjo scale (58) for causality as shown in Supplementary Table 1. Naranjo scale was used for causality assessment of the case reports, that allows categorical classification of adverse events as “definite,” “probable,” “possible,” or “doubtful” based on the answers to 10 questions. One investigator KE carried out the assessment and NO and NE randomly re-checked it.

## Results

### Patient Characteristics

Patient characteristics are described in Table 3. One hundred and eighteen cases were identified in 49 articles. The median age of cases was 54 years (range 32–85 years). The majority of cases were males (*n* = 73, 61.8%). Most patients had melanoma (57.6%) and lung cancer (26.3%). Other cancer sites included prostate (*n* = 1), bladder (*n* = 1), glioblastoma multiforme (*n* = 1), renal cell carcinoma (*n* = 4), and others (*n* = 10). Fifty three (44.9%) cases were labeled as stage 4, two cases as stage 3, one case as locally advanced disease, while in 61 (51.7%) cases, the stage of cancer was not mentioned. Twenty one (17.8%) cases were confirmed to have bone metastasis, while 55 (46.6%) cases did not have bone metastasis and no data were mentioned for the remaining 42 (35.5%) cases.

**Table 3:** Characteristics of the described patients in the eligible case reports.

**References**	**Therapeutic agent**	**Age**	**Gender**	**Stage of the disease**	**Bone metastasis Y, N, or NA**	**How many line/s of therapy before ICPi**	**History of radiotherapy Y or N**	**History of autoimmune or hematological disorder/s before ICPi**	**Bone marrow Biopsy done Y or N**	**Grade of Hem-IRAEs according to the (CTCAE)**
(10) Case a Case b	Pembrolizumab	34 51	M F	4 locally advanced	NA N	4 lines None	Y N	NA None	Y Y	NA NA
(11)	Pembrolizumab	73	M	4	Y	3 lines	Y	IFN-α Hashimoto thyroiditis mild thrombocytopenia	N	NA
(12)	Pembrolizumab	52	F	4	N	1 line	N	None	Y	4
(13)	Pembrolizumab	52	F	4	N	2 lines	Y	Autoimmune hepatitis	Y	NA
(14)	Pembrolizumab	73	F	4	NA	1 line	NA	Autoimmune myositis (in remission)	N	4
(15)	Pembrolizumab	76	M	4	NA	NA	NA	NA	Y	NA
(16)	Pembrolizumab	67	M	4	N	2 lines	Y	NA	N	NA
(17)	Pembrolizumab	82	M	3a	N	1 line	N	Chronic anemia	Y	NA
(18)	Pembrolizumab	79	F	4	Y	1 line	Y	None	N	NA
(19)	Pembrolizumab	78	M	4	NA	1 line	NA	NA	N	NA
(20)	Nivolumab	73	M	4	N	2 lines	Y	Moderate macrocytic anemia and mild thrombocytopenia	Y	4
(21)	Nivolumab	56	M	4	N	1 line	NA	None	Y	NA
(22)	Nivolumab	32	M	4	Y	3 lines	Y	Mild ITP	N	NA
(23)	Nivolumab	78	M	4	Y	1 line	N	Early stage lymphoma (in remission)	Y	4
(24)	Nivolumab	62	M	4	NA	2 lines	NA	Asymptomatic Hashimoto's thyroiditis	Y	NA
(25)	Nivolumab	79	F	4	N	1 line	N	NA	N	4
(4)	Nivolumab	74	F	4	Y	1 line	Y	Ulcerative colitis (in remission)	Y	NA
(26) Case a Case b	Nivolumab	73 74	M M	Both cases were stage 4	N N	4 lines 3 lines	N N	None Treated intermediate grade follicular lymphoma	N Y	4 4
(8) Case a Case b Case c	Nivolumab	73 70 78	F M M	All 3 cases were stage 4	N N Y	2 lines 3 lines 1 line	Y N Y	None None None	Y Y Y	Sever cytopenias, grade 3 or higher
(27)	Nivolumab	85	M	4	N	2 lines	N	None	N	NA
(28)	Nivolumab	82	M	4	Y	2 lines	Y	CLL	N	NA
(29)	Nivolumab	70	M	4	NA	1 line	N	NA	N	NA
(30)	Nivolumab	57	F	4	N	2 lines	Y	None	Y	4
(31)	Nivolumab	70	F	4	N	1 line	Y	None	Y	NA
(32)	Nivolumab	73	M	4	NA	NA	NA	None	Y	NA
(33)	Nivolumab	63	F	4	NA	3 lines	NA	None	Y	NA
(34)	Nivolumab	68	M	4	NA	1 line	NA	None	N	NA
(35)	Ipilimumab	77	F	4	N	2 lines	N	History of regressive thyroiditis	Y	4
(36)	Ipilimumab	NA	NA	4	NA	Heavily pretreated	NA	NA	NA	4
(37)	Ipilimumab	NA	NA	4	NA	NA	NA	NA	NA	4
(38)	Ipilimumab	57	M	4	Y	1 line	Y	None	Y	4
(39)	Ipilimumab	54	M	4	N	1 line	N	None	Y	4
(40)	Ipilimumab	42	F	4	N	5 lines	Y	None	Y	4
(41)	Ipilimumab	35	M	4	Y	1 line	Y	None	Y	4
(42) Case a Case b Case c	Ipilimumab	68 49 70	F F M	All 3 cases were stage 4	N Y N	1 line 1 line 1 line	NA Y N	NA NA NA	N Y Y	NA
(43)	Ipilimumab	54	M	3b	N	None	N	None	Y	NA
(44)	Ipilimumab	74	F	4	NA	1 line	NA	NA	Y	4
(45)	Ipilimumab	42	M	4	Y	3 lines	N	None	N	NA
(46)	Ipilimumab	55	M	4	N	3 lines	N	None	Y	NA
(47)	Ipilimumab	52	F	4	N	1 line	Y	Indolent lymphoplasmocytic lymphoma	Y	NA
(48)	Durvalumab	39	M	4	NA	None	NA	None	Y	4
(49)	Avelumab	77	M	4	N	None	N	B12 and folic acid deficiency	Y	4
(50) Case a Case b	Ipilimumab and nivolumab	47 45	F F	2b 4		1 line None	N N	NA Thrombocytopenia	Y N	4
(51)	Ipilimumab plus nivolumab	48	F	4	NA	NA	NA	NA	Y	NA
(52)	Ipilimumab and nivolumab	43	F	4	N	NA	Y	None	N	NA
(53) Case a Case b Case c Case d	Case a: Ipilimumab Case b: Pembrolizumab Case c: Pembrolizumab Case d: Ipilimumab and nivolumab	64 58 62 64	M F M M	4 4 4 4	N N NA NA	3 lines NA 1 line NA	NA NA Y NA	None None NA None	N Y N N	NA
(54) (26 cases)	Pembrolizumab (*n* = 17) Nivolumab (*n* = 7) Durvalumab (*n* = 2)	58^#^	19 M 7 F	Miscellaneous	NA	Median: 1 [Range 0–7]	NA	NA	NA	NA
(55)	Ipilimumab and nivolumab	51	M	4	N	1 line	Y	None	Y	4
(56) (35 cases)	Nivolumab (*n* = 20) Pembrolizumab (*n* = 14) atezolizumab (*n* = 1)	65^**^	21 M 14 F	Miscellaneous	10 Y 25 N	Median: 2 [Range 1–3]	16 Y 19 N	The 3 cases had a history of b-c 2	Y	Grade 2 *n* = 3 Grade 3 *n* = 5 Grade 4 *n* = 25 Grade 5 *n* = 2

# age range (3–87),

** *age range (51–75). Y, yes; N, no; NA, not available; ITP, immune thrombocytopenia; IFN-α, Interferon alpha; CTCAE, Common Terminology Criteria of Adverse Events*.

Thirty seven (31.3%) cases were treated with radiotherapy, while 38 (32.2%) cases did not receive radiotherapy and no history of exposure to radiotherapy in 43 (36.5%) cases.

Heavily pretreated patients were defined as patients who previously received two or more lines of treatment; 56 (47.5%) cases were heavily pretreated; 50 (42.4) cases received only one previous line of treatment; 5 cases were treatment naïve. With respect to history of autoimmune or hematological disorders before the use of ICPis; no data was provided in 73 (61.8%) cases, while 18 (15.3%) cases had history of either autoimmune or hematological disorder before ICPis usage, while 27 (22.9%) cases did not have history. A bone marrow biopsy was done to confirm the Hem-irAEs in 71 (61.2%) cases, but it was not done in 19 (16.1%) cases. The grade of Hem-irAEs was labeled as grade 2 in 3 cases, grade 3 in 5 cases, grade 4 in 50 (42.3%) case, and grade 5 in 2 cases.

### Nivolumab

Seventeen case studies (out of 49) reported Hem-irAEs with nivolumab in 20 cases (13 lung cancer, 5 melanoma, 1 cutaneous squamous cell carcinoma, and 1 glioblastoma). Anemia was reported in 7 cases; two had aplastic anemia and five had hemolytic anemia. Thrombocytopenia was reported in five cases. Bone marrow failure was reported in three cases, pancytopenia in one case, neutropenia in one case, red cell aplasia in one case, hemophagocytic syndrome in one case, agranulocytosis in one case and acquired hemophilia A in one case.

Treatment was reported for all patients. Resolution of the adverse events was reported in 11 cases (55%) and treatment was ineffective in 8 cases (40%). One case showed partial and transient response to treatment. In the 11 cases that showed response, the most common treatment for Hem-irAEs was IV corticosteroids, however, IV romiplostim, platelets transfusion, IVIG, and oral steroids were used. Many patients had to discontinue nivolumab with the treatment used.

Another two-case series reported Hem-irAEs with nivolumab in 27 cases. An increase in the absolute eosinophil count was reported by Bernard-Tessier et al. (54). No treatment was mentioned in this report. Delanoy et al. (56) reported neutropenia, anemia, thrombocytopenia, pancytopenia, bicytopenia, pure red cell aplasia with nivolumab, pembrolizumab, and atezolizumab. Twenty one patients had resolved symptoms with oral steroids, IV steroids, IVIG, and rituximab.

### Ipilimumab

Fourteen articles reported Hem-irAEs with ipilimumab in 16 cases (15 melanomas and one with prostate cancer). The adverse events reported were neutropenia (5 cases), pancytopenia (3 cases), leukopenia (3 cases), thrombocytopenia (2 cases), anemia (2 cases), and 5 cases showed one of the following adverse events: agranulocytosis, lymphocytosis, hemophagocytic syndrome, acquired hemophilia A, and red cell aplasia. Eleven cases (68.75%) recovered after treatment. Steroids (8 cases) and IVIG (7 cases) were the most commonly used treatments.

### Pembrolizumab

Twelve reports described Hem-irAEs with pembrolizumab in 13 cases (7 melanomas, 4 lung cancer, and 1 bladder cancer). In these cases, hemolytic anemia was reported in five cases and thrombocytopenia in two cases. Neutropenia, pancytopenia, red cell aplasia, hemophagocytic lymphohistiocytosis, and Evan's syndrome were reported in one case each. Adverse events were resolved in 11 cases. Steroids (whether IV or oral) were used in all the managed cases, and IVIG was used in five cases.

### Combination of Ipilimumab—Nivolumab

This combination of ipilimumab and nivolumab, used to treat metastatic melanoma, was associated with Hem-irAEs in 6 cases (5 reports). Thrombocytopenia, aplastic anemia, and hemolytic anemia were reported in two cases each. The adverse events were resolved in 5 cases. One case died with refractory aplastic anemia. Rituximab was a commonly used treatment; one patient with thrombocytopenia recovered after 4 doses of rituximab following IVIG failure. The second occurrence of hemolytic anemia in one of the cases resolved with rituximab use.

### Durvalumab

A fatal allo- and immune-mediated thrombocytopenia was reported with durvalumab use in one NSCLC case. Platelet transfusion, polyvalent immunoglobulins and steroid treatments were used to treat the patient without improvement.

### Avelumab

One patient with metastatic Merkel cell carcinoma developed lethal immune thrombocytopenia (ITP) after avelumab administration. Treatments with steroids and IVIG were ineffective and the patient died after 1 month from initial diagnosis.

Concerning the treatment of Hem-irAEs reported, steroids were the most commonly used (80/118, 67.7%), with a failure rate of (16/80 = 20%) out of 118 cases. Other treatment options included IVIG, rituximab, and combination of the three options at varying doses.

#### Quality Assessment

Table 2 shows quality assessment of the extracted citations using Pierson-5. The number of case reports is based on five domains: uniqueness, documentation, objectivity, interpretation, and educational value. Every domain is scored with 2 points, the upper score is 10 points. Naranjo scale was used for causality assessment of the case reports, that allows categorical classification of adverse events as “definite,” “probable,” “possible,” or “doubtful” based on the answers to 10 questions.

Fifty-four case reports were retrieved from the literature and assessed. Out of the 54 reports, 5 (9.2%) could not be assessed, since the data presented were insufficient for assessment for 4 of them, while 1 study was an observational study. Seven cases (12.9%) were rated as “of insufficient quality for publication” because they scored 5 or less. The second case reported in Shiuan et al. (50) got zero score in the five domains. Twenty-six studies (48.1%) were assessed as “reader should be cautious about validity and clinical value of the report” because they scored 7–8. Twenty-one cases (38.8%) were rated as “likely to be a worthwhile contribution to the literature” as they scored 9–10.

#### Causality Assessment

Eight studies were ranked as “possible” adverse drug reaction, scoring 3 (one study) and 4 (7 studies). Two studies were not assessed because their data were insufficient. Sixteen studies were ranked as “probable” adverse drug reaction as they scored between 5 and 8. No cases were ranked as “definite” or “doubtful” adverse drug reaction.

For pembrolizumb case reports (13 reports), 8 of them (61.5%) were assessed as probable Hem-irAEs. Next to pembrolizumab, nivolumab (20 reports), 12 of which (60%) were assessed as probable, then comes ipilimumab (14 reports), 8 of which (57%) were assessed as probable. For the combination of ipilimumab and nivolumab (6 reports), 3 of them (50%) were assessed as probable. Finally, only one case report was assessed for durvalamab where the causality assessment yielded as a possible Hem-irAEs.

## Discussion

Immunotherapeutics are increasingly used in cancer patients. However, adverse events can limit their use and may result in serious adverse outcomes, including death. While some adverse events have been well-described in clinical trials (e.g., dermatitis and colitis), other inflammatory and autoimmune manifestations are reported. Case reports can provide vital clues and signals to identify rare but serious events and can generate hypotheses that can direct ongoing scientific research. We conducted a systematic review of case reports/series of patients treated with checkpoint blockade to identify the scope of rare Hem-irAEs that may occur with these therapies. We included publications that had adequate description of the clinical manifestations of the patients reported.

This systematic review showed thrombocytopenia, hemolytic and aplastic anemias as the most commonly associated with ICPis use, i.e., nivolumab, ipilimumab, and pembrolizumab. Less reported adverse events included agranulocytosis and neutropenia. Steroids (either intravenous or oral) were commonly used to treat these adverse events with frequent success. Other strategies used IVIG, rituximab and transfusion of blood components.

The mechanisms of the recorded adverse events in the included articles remain elusive. The most plausible theory is activation of T-cells, leading to the secretion of different cytokines from T-helper cells and consequent tissue infiltration with cluster of differentiation 8 (CD8) T-cytotoxic cells (59). Another suggested mechanism was immune-mediated dysfunction in hematopoietic cell maturation and proliferation, yet, the exact intermediate mechanism is unknown (20). The response to steroids in the majority of these cases potentiates the theory of immune-mediated mechanisms that occur centrally (in the bone marrow) or peripherally (in the circulation).

We used the Naranjo scale to infer causality of the reported adverse event to the used ICPi drug. Although data were not available for some reports, we showed possible or probable causality in several included reports. In some of these reports, the ICPi was the only new treatment introduced and the events diminished after the drug withdrawal. Further, the temporal relationship between ICPis administration and the occurrence of the adverse effect implicates these drugs. Hem-irAEs are known to occur within 12 to 16 weeks of treatment initiation (60).

As reflected from the causality assessment results, the majority of cases reported were “probable”; being at the near top of the causality continuum of the Naranjo scale (just before definite). Consequently, the association between ICPis and Hem-irAEs cannot be ignored.

This review provides insights into the proper management strategies for Hem-irAEs. Previously, it was thought that cancer patients receiving immunotherapy should not receive immunosuppressive drugs. This view has significantly changed over the past few years and the use of immunosuppressive agents has been proven not to impair the efficacy of ICPis (61). Corticosteroids should be the first resource and some reports highlighted the benefit of high dose steroids therapy. In grade 3/4 adverse events, the ICPis should be discontinued and steroids can later be tapered off in 4 to 6 weeks with close monitoring of blood counts (7). Other immunosuppressive drugs as IVIG, rituximab or tumor necrosis factor antagonists may also be effective. In case the immunosuppressive therapy is prolonged, immunization against pneumocystis is recommended (4).

Definitions of the side effects in the registries of rare events are poor. Therefore, we focused on the qualitative features such as demographic characteristics of patients, diagnosis and management. We did not perform quantitative analysis of these case reports because risk analysis was not possible. Randomized clinical trials were not related to our objective and were excluded in this systematic review. Limiting the inclusion criteria to studies published in English was challenging. However, a former analysis showed that this language limitation does not usually alter the study results (62).

Future case reports/series should follow a standardized approach in reporting their patients characteristics and findings. Further attention should be given to Hem-irAEs in ICPis randomized controlled trials to provide higher quality data in this regard. Moreover, the mechanisms of these adverse events should be investigated on the molecular and cellular levels to specify more effective pharmacological interventions. The management of Hem-irAEs in patients receiving ICPis needs evidence-based guidelines to inform future practice and research in this area.

Concerning the factors that may have predisposed patients to the adverse effects, there was no clear pattern for age. Patients characteristics were heterogenous for age with high interpatient variability with median age of 54 years and wide range 32–85 years. For gender, most patients were males (*n* = 73, 61.8%); although the percentage is not conclusive, it warrants further investigations and more research.

There was no predictor for the response to treatment. However, steroids were the most commonly used option. This can be explained secondary to its relative availability, low cost, and physicians' experience compared to other options. Furthermore, steroid was not always successful (20% failure rate) which implies seeking other treatment options and keeping patients on steroids for Hem-irAEs closely monitored.

## Conclusion

Although rare, Hem-irAEs are serious adverse events that may be associated with checkpoint blockade therapy. Depending on the grade of the adverse event, the ICPi therapy may be discontinued and steroid therapy should be initiated. Steroids were the most commonly management strategy with considerable failure rate. There were no detected underlying factors predicting the outcome to steroid therapy. Other promising management strategies for some events include IVIG, rituximab, and transfusion of blood components.

## Future Research Recommendation

Further research should focus on the plausible mechanisms contributing to these adverse events, to develop more specific management strategies.

## Data Availability Statement

Datasets are available on request from the authors.

## Author Contributions

NO and NE extracted eligible articles. KE-F conducted initial screening of the eligible articles. Any conflict was solved by KE-F. The assessment was carried out by KE-F. A random sample was cross checked by NO and NE. AA, MY, AH, and SE contributed to the analysis. DJ, AA, AB, and AN contributed to writing of the manuscript and discussion. SD contributed to the discussion and reviewing the scientific background. All authors approved the article for submission.

## Conflict of Interest

The authors declare that the research was conducted in the absence of any commercial or financial relationships that could be construed as a potential conflict of interest.

## Abbreviations

Hem-irAEs: Hematological Immune-Related Adverse Events
ICPis: Immune Checkpoint Inhibitors
ITP: Immune Thrombocytopenia
IVIG: Intravenous Immunoglobulins
ESMO: The European Society for Medical Oncology
CTLA4: Cytotoxic T-Lymphocyte-Associated Protein 4
PD-1: Programmed Cell Death Protein-1
SCLC: Small Cell Lung Carcinoma
NSCLC: Non-Small Cell Lung Cancer
ORRs: Objective Response Rates
PRISMA: Preferred Reporting Items for Systematic Reviews and Meta-Analyses
CD8: Cluster of Differentiation 8
IVATG: Intravenous Anti-thymocyte Globulin
CSF: Colony Stimulating Factor
G-CSF: Granulocyte Colony Stimulating Factor
GM-CSF: Granulocyte-Macrophage Colony Stimulating Factor
RBC: Red Blood Cells
AEC: Absolute Eosinophil Count
AHA: Autoimmune Hemolytic Anemia
IFN-α: Interferon alpha
CTCAE: Common Terminology Criteria of Adverse Events.
