# Supplementary material for: Diagnosis and Management of Hematological Adverse Events Induced by Immune Checkpoint Inhibitors: A Systematic Review
Source: Front Immunol. 2020 Oct 21;11:1354. doi: 10.3389/fimmu.2020.01354 (PMC7640759; doi:10.3389/fimmu.2020.01354)
Supplement: Supplementary file 1 [file Table_1.docx]

Supplementary Tables

Supplementary Table 1: List of case reports studies with Naranjo scale score and Pierson-5 evaluation scheme results

| Case report | Drug involved | Naranjo score | Pierson-5 score | Pierson-5 score interpretation |
| --- | --- | --- | --- | --- |
| Le roy et al. 2015 – case A | Pembrolizumab | 6 (probable) | 7 | Caution^a^ |
| Le roy et al. 2015 – case B | Pembrolizumab | 8 (Probable) | 7 | Caution |
| Pföhler Claudia et al. 2017 | Pembrolizumab | 3 (possible) | 4 | Insufficient^b^ |
| Atwal Dinesh et al. 2017 | Pembrolizumab | 5 (probable) | 10 | Worthwhile^c^ |
| Nair R et al. 2016 | Pembrolizumab | 6 (probable) | 7 | Caution |
| Barbacki Ariane et al. 2018 | Pembrolizumab | 4 (possible) | 4 | Insufficient |
| Shah D et al 2017 | Pembrolizumab | 5 (probable) | 8 | Caution |
| Lott Anthony et al. 2015 | Pembrolizumab | 4 (possible) | 8 | Caution |
| Ogawa Kenta et al. 2018 | Pembrolizumab | 7 (probable) | 9 | Worthwhile |
| Robilliard B et al. 2018 | Pembrolizumab | 4 (possible) | 8 | Caution |
| Sherbeck J et al 2018 | Pembrolizumab | 4 (possible) | 8 | Caution |
| Inadomi et al. 2016 | Nivolumab | 3 (possible) | 5 | Insufficient |
| Tokumo Kentaro et al. 2018 | Nivolumab | 3 (Possible) | 6 | Caution |
| S.J. Bagley et al. 2016 | Nivolumab | 8 (Probable) | 9 | Worthwhile |
| Karakas Y et al. 2017 | Nivolumab | 6 (probable) | 8 | Caution |
| Jotatsu, T et al. 2018 | Nivolumab | 5 (Probable) | 9 | Worthwhile |
| Kanameishi S et al. 2015 | Nivolumab | 6 (probable) | 8 | Caution |
| Tabchi S et al. 2016 | Nivolumab | 6 (probable) | 8 | Caution |
| Turgeman et al. 2017 - case A | Nivolumab | 3 (possible) | 9 | Worthwhile |
| Turgeman et al. 2017 - case B | Nivolumab | 6 (Probable) | 9 | Worthwhile |
| Michot J.-M et al. 2017 – case A | Nivolumab | 5 (Probable) | 9 | Worthwhile |
| Michot J.-M et al. 2017 – case B | Nivolumab | 5 (Probable) | 9 | Worthwhile |
| Michot J.-M et al. 2017 – case C | Nivolumab | 5 (Probable) | 9 | Worthwhile |
| Kong et al. 2016 | Nivolumab | 4 (possible) | 10 | Worthwhile |
| Schwab et al. 2016 | Nivolumab | 7 (probable) | 7 | Caution |
| Palla et al. 2016 | Nivolumab | 6 (probable) | 9 | Worthwhile |
| Comito et al. 2017 | Nivolumab | 4 (possible) | 9 | Worthwhile |
| Yuki et al. 2017 | Nivolumab | 4 (possible) | 9 | Worthwhile |
| Deltombe C et al. 2017 | Nivolumab | Data presented not enough for assessment | | |
| Takeshita M et al. 2016 | Nivolumab | 4 (possible) | 9 | Worthwhile |
| Kato et al. 2018 | Nivolumab | 6 (probable) | 9 | Worthwhile |
| DuRusquec Pauline et al. 2014 | Ipilimumab | 7 (probable) | 5 | Insufficient |
| Di Giacomo et al. 2011 | Ipilimumab | Data presented not enough for assessment | | |
| Zimmer et al. 2015 | Ipilimumab |  |  |  |
| Ahmad et al. 2011 | Ipilimumab | 3 (Possible) | 5 | Insufficient |
| Kopecký J et al. 2015 | Ipilimumab | 2 (Possible) | 6 | Caution |
| Akhtari M et al. 2009 | Ipilimumab | 6 (probable) | 10 | Worthwhile |
| Woźniak S et al. 2015 | Ipilimumab | 4 (possible) | 7 | Caution |
| Simeone E et al. 2014- Case A | Ipilimumab | 5 (probable) | 6 | Caution |
| Simeone E et al. 2014- Case B | Ipilimumab | 4 (possible) | 6 | Caution |
| Simeone E et al. 2014- Case C | Ipilimumab | 4 (possible) | 6 | Caution |
| Ban-Hoefen M et al. 2016 | Ipilimumab | 4 (possible) | 8 | Caution |
| Wei G et al. 2012 | Ipilimumab | 6 (probable) | 10 | Worthwhile |
| Delyon J et al. 2011 | Ipilimumab | 6 (probable) | 9 | Worthwhile |
| Gordon et al. 2009 | Ipilimumab | 6 (probable) | 10 | Worthwhile |
| Michot J. et al. 2018 | Ipilimumab | 6 (probable) | 8 | Caution |
| Leroy L et al. 2018 | Durvalumab | 3 (Possible) | 7 | Caution |
| Kratzsch D et al. 2018 | Nivolumab | 3 (possible) | 2 | Insufficient |
| Shiuan et al. 2017- Case A | Ipilimumab and Nivolumab | 4 (possible) | 10 | Worthwhile |
| Shiuan et al. 2017- Case B | Ipilimumab and Nivolumab | 4 (possible) | 0 | Insufficient |
| Helgadottir et al. 2017 | Ipilimumab and Nivolumab | 3 (possible) | 6 | Caution |
| Khan Uqba et al. 2017 | Ipilimumab and Nivolumab | 8 (probable) | 10 | Worthwhile |
|  |  |  |  |  |
| Sun et al. 2017- Case A | ipilimumab | 5 (Probable) | 6 | Caution |
| Sun et al. 2017- Case B | Pembrolizumab | 7 (Probable) | 6 | Caution |
| Sun et al. 2017- Case C | Pembrolizumab | 7 (Probable) | 6 | Caution |
| Sun et al. 2017- Case D | Ipilimumab and Nivolumab | 7 (Probable) | 6 | Caution |
| Bernard-Tessier et al. 2017- 26 Cases | Pembrolizumab, Nivolumab Durvalumab | Data presented are not enough for assessment | | |
| Meyers et al. 2018 | Ipilimumab and Nivolumab | 6 (Probable) | 9 | Worthwhile |
| Delanoy et al. 2019 - 35 cases | Observational study not including data for assessment | | | |

*a: reader should be cautious about validity and clinical value of report*

*b: report is of insufficient quality for publication*

*c: report is likely to be a worthwhile contribution to the literature*
