# Supplementary material for: Diagnosis and Management of Hematological Adverse Events Induced by Immune Checkpoint Inhibitors: A Systematic Review
Source: Front Immunol. 2020 Oct 21;11:1354. doi: 10.3389/fimmu.2020.01354 (PMC7640759; doi:10.3389/fimmu.2020.01354)
Supplement: Supplementary file 2 [file Data_Sheet_1.pdf]

Search terms

Plumbed

("Anemia"[Mesh]) AND "Ipilimumab"[Mesh]
((("Nivolumab"[Mesh]) AND "Anemia"[Mesh])) AND ipilimumab
("Nivolumab"[Mesh]) AND "Anemia"[Mesh]
("pembrolizumab" [Supplementary Concept]) AND "Anemia"[Mesh]
("Antineoplastic Agents, Immunological"[Mesh]) AND "Anemia"[Mesh]
("atezolizumab" [Supplementary Concept]) AND "Anemia"[Mesh]
("Anemia"[Mesh]) AND "avelumab" [Supplementary Concept]
("Anemia"[Mesh]) AND "durvalumab" [Supplementary Concept]

("atezolizumab" [Supplementary Concept]) AND "Pancytopenia"[Mesh]
("durvalumab" [Supplementary Concept]) AND "Pancytopenia"[Mesh]
("avelumab" [Supplementary Concept]) AND "Pancytopenia"[Mesh]
("Pancytopenia"[Mesh]) AND "Ipilimumab"[Mesh]
("Nivolumab"[Mesh]) AND "Pancytopenia"[Mesh]
("Antineoplastic Agents, Immunological" [Pharmacological Action]) AND "Pancytopenia/complications"[Mesh
("Pancytopenia"[Mesh]) AND "pembrolizumab" [Supplementary Concept]
("Pancytopenia"[Mesh]) AND "Antineoplastic Agents, Immunological" [Pharmacological Action]

("Neutropenia"[Mesh]) AND "pembrolizumab" [Supplementary Concept]
("Neutropenia"[Mesh]) AND "nivolumab" [Mesh]
("Neutropenia"[Mesh]) AND "Ipilimumab"[Mesh]
(("Neutropenia"[Mesh]) AND "Nivolumab"[Mesh]) AND "Ipilimumab"[Mesh]
("Neutropenia"[Mesh]) AND "atezolizumab" [Supplementary Concept]
("Neutropenia"[Mesh]) AND "avelumab" [Supplementary Concept]
("Neutropenia"[Mesh]) AND "durvalumab" [Supplementary Concept]
("Antineoplastic Agents, Immunological"[Mesh]) AND "Neutropenia"[Mesh]

("Leukopenia"[Mesh]) AND "pembrolizumab" [Supplementary Concept]
("Antineoplastic Agents, Immunological"[Mesh]) AND "Leukopenia"[Mesh]
("Leukopenia"[Mesh]) AND "Nivolumab"[Mesh]
("Leukopenia"[Mesh]) AND "atezolizumab" [Supplementary Concept]
("Leukopenia"[Mesh]) AND "avelumab" [Supplementary Concept]
("durvalumab" [Supplementary Concept]) AND "Leukopenia"[Mesh]

("pembrolizumab" [Supplementary Concept]) AND "Thrombocytopenia"[Mesh]
("Antineoplastic Agents, Immunological"[Mesh]) AND "Thrombocytopenia"[Mesh]
("Thrombocytopenia"[Mesh]) AND "Nivolumab"[Mesh]
("Thrombocytopenia"[Mesh]) AND "atezolizumab" [Supplementary Concept]
("Thrombocytopenia"[Mesh]) AND "avelumab" [Supplementary Concept]
("Thrombocytopenia"[Mesh]) AND "durvalumab" [Supplementary Concept]

web of science

TS= (pembrolizumab* near thrombocytopenia*) or TS= (pembrolizumab* near pancytopenia*) or TS= (pembrolizumab* near neutropenia*) or TS= (pembrolizumab* near leukopenia*) or TS= (pembrolizumab* near haemophilia*)"

"TS= (nivolumab*near thrombocytopenia*) or TS= (nivolumab* near pancytopenia*) or TS= (nivolumab* near neutropenia*) or TS= (nivolumab* near leukopenia*) or TS= (nivolumab* near haemophilia*)"

"TS= (ipilimumab*near thrombocytopenia*) or TS= (ipilimumab* near pancytopenia*) orTS= (ipilimumab* near neutropenia*) or TS= (ipilimumab* near leukopenia*) or TS= (ipilimumab* near haemophilia*)"

"TS= (avelumab*near thrombocytopenia*) or TS= (avelumab* near pancytopenia*) or TS= (avelumab* near neutropenia*) or TS= (avelumab* near leukopenia*) or TS= (avelumab* near haemophilia*)"

"TS= (atezolizumab* near thrombocytopenia*) or TS= (atezolizumab* near pancytopenia*) or TS= (atezolizumab* near neutropenia*) or TS= (atezolizumab* near leukopenia*) or TS= (atezolizumab* near haemophilia*)"

"TS= (durvalumab* near thrombocytopenia*) or TS= (durvalumab* near pancytopenia*) or TS= (durvalumab* near neutropenia*) or TS= (durvalumab* near leukopenia*) or TS= (durvalumab* near haemophilia*)"

OVID SEARCH

Immune checkpoint inhibitors and pancytopenia).mp. [mp=title, abstract, full text, caption text]

(Immune checkpoint inhibitors and anemia).mp. [mp=title, abstract, full text, caption text]

(Immune checkpoint inhibitors and thrombocytopenia).mp. [mp=title, abstract, full text, caption text]

(Immune checkpoint inhibitors and neutropenia).mp. [mp=title, abstract, full text, caption text]

immune checkpoint inhibitors and leukopenia).mp. [mp=title, abstract, full text, caption text]
